# WHO calls for urgent action by countries for achieving medication without harm

**Published:** 2022-10

**16 September 2022** - Globally, half of all preventable harm in medical care is medication related, a quarter of which is severe or life-threatening. In the lead up to World Patient Safety Day on 17 September 2022, WHO is emphasizing the global burden of medication harm. The elderly population is one of the most at-risk groups of medication harm, especially those taking multiple medications. High rates of medication-related harm are also seen in surgical care, intensive care and emergency medicine.

“Medicines are powerful tools for protecting health. But medicines that are wrongly prescribed, taken incorrectly or are of poor quality, can cause serious harm,” said Dr Tedros Adhanom Ghebreyesus, WHO Director-General. “Nobody should be harmed while seeking care.”

Unsafe medication practices and medication errors are one of the main causes of injury and avoidable harm in health-care systems across the world. The global cost associated with medication errors has been estimated at US$42 billion annually. Medication errors happen due to systemic issues and/or human factors such as fatigue, poor environmental conditions or staff shortages which affect prescribing, transcribing, dispensing, administration and monitoring practices. These errors can result in severe harm, disability and even death.

World Patient Safety Day aims to increase understanding among and engagement of the public and encourage countries to promote safety in health care. This year has a particular focus on medication safety with the slogan ‘Medication Without Harm’. The campaign will also see the consolidation of the ongoing WHO Global Patient Safety Challenge: Medication Without Harm, with the aim of reducing avoidable medication-related harm globally.

WHO is advocating for urgent improvement in strategies to reduce medication-related harm in key risk areas. Furthermore, it is working with partners to develop a set of medication safety technical resources, including a policy brief and medication safety solutions such as medication safety for look-alike sound-alike (LASA) medicines. LASA medicines may look or sound similar to each other, either by their generic name, or brand name. They might have similar packaging, similar-sounding names, or similar spellings.

Flaws in the systems for prescription are a big contributor to medication-related harm, alongside human error. Evidence has shown that more than half of all medication harm occurs at the stage when medicines are prescribed and when they are being taken by patients due to inadequate monitoring. The highest risk category for medication-related harm is antibiotics, but medicines such as sedatives, anti-inflammatories and heart and blood pressure medication also pose significant risks.

WHO is calling on stakeholders to continue efforts to reduce medication-related harm, develop strategies and structures to improve medication safety at local, national, regional and global levels, and make a pledge to adopt the Medication Without Harm Challenge.

Available from: https://www.who.int/news/item/16-09-2022-who-calls-for-urgent-action-by-countries-for-achieving-medication-without-harm

